# Possible Transmission of Severe Acute Respiratory Syndrome Coronavirus 2 (SARS-CoV-2) in a Public Bath Center in Huai’an, Jiangsu Province, China

**DOI:** 10.1001/jamanetworkopen.2020.4583

**Published:** 2020-03-30

**Authors:** Chao Luo, Lun Yao, Li Zhang, Mengchu Yao, Xiaofei Chen, Qilong Wang, Hongbing Shen

**Affiliations:** 1 Department of Central Laboratory, The Affiliated Huai’an No. 1 People’s Hospital, Nanjing Medical University, Huai’an, China; 2 Department of Infection Diseases, Huai’an No. 4 Hospital, Huai’an, China; 3 Department of Clinical Oncology, The Affiliated Huai’an No. 1 People’s Hospital, Nanjing Medical University, Huai’an, China; 4 Department of Epidemiology and Biostatistics, Jiangsu Key Lab of Cancer Biomarkers, Prevention, and Treatment, Collaborative Innovation Center for Cancer Medicine, School of Public Health, Nanjing Medical University, Nanjing, China

## Abstract

This case series reports a cluster-spreading event in Huai’an, Jiangsu Province, China, in which a patient with coronavirus disease 2019 (COVID-19) may have transmitted severe acute respiratory syndrome coronavirus 2 (SARS-CoV-2) to 8 healthy individuals.

## Introduction

In December 2019, a novel pneumonia named coronavirus disease 2019 (COVID-19), caused by severe acute respiratory syndrome coronavirus 2 (SARS-CoV-2), emerged in Wuhan, China, and has since spread to 25 countries. Current reports show that SARS-CoV-2 is closely related to severe acute respiratory syndrome coronavirus (SARS-CoV) and Middle East respiratory syndrome coronavirus (MERS-CoV)^1,2^ and that it has a greater transmissibility than other coronaviruses. The confirmed transmission modes of SARS-CoV-2 include respiratory droplets and physical contact, and the incubation period for the virus is approximately 3 to 7 days, but it can be as long as 24 days.^3^ In this case series, we report a cluster-spreading event in Huai’an (700 km northeast of Wuhan) in Jiangsu Province, China, in which a patient with SARS-CoV-2 may have transmitted the virus to 8 other healthy individuals via bathing in a public bath center.

## Methods

Data were collected from Huai’an No. 4 Hospital of Jiangsu Province, China. A total of 9 patients who had been to the same bath center were hospitalized and enrolled from January 25, 2020, to February 10, 2020. Throat swab samples were collected, and SARS-CoV-2 was detected using a quantitative reverse transcription–polymerase chain reaction assay. Computed tomography and hematological examinations were performed for auxiliary diagnoses. Data were analyzed with Prism version 7.00 (GraphPad). This study was approved by the ethics committee of Huai’an No. 4 Hospital, and written informed consent was obtained from all patients. This study followed the reporting guideline for case series. No prespecified threshold for statistical significance was set.

## Results

The bath center for men was approximately 300 m^2^, with temperatures from 25 to 41 °C and humidity of approximately 60%. It contained a swimming pool, showers, and sauna. The first patient (patient 1) had traveled to Wuhan. He went to the bath center and showered on January 18, 2020. He started experiencing a fever on January 19, 2020, and was diagnosed with COVID-19 on January 25, 2020. The next 7 patients showered, used the sauna, and swam in the same center on January 19 (patients 2, 3, and 4), January 20 (patient 5), January 23 (patients 6 and 7), and January 24 (patient 8). The symptoms associated with COVID-19, including fever, cough, headache, and chest congestion, appeared between 6 and 9 days after visiting the bath center. Patient 9 was working in the bath center and experienced onset on January 30. Infection in all patients was confirmed by positive reverse transcription–polymerase chain reaction assay results (Figure).

**Figure.**
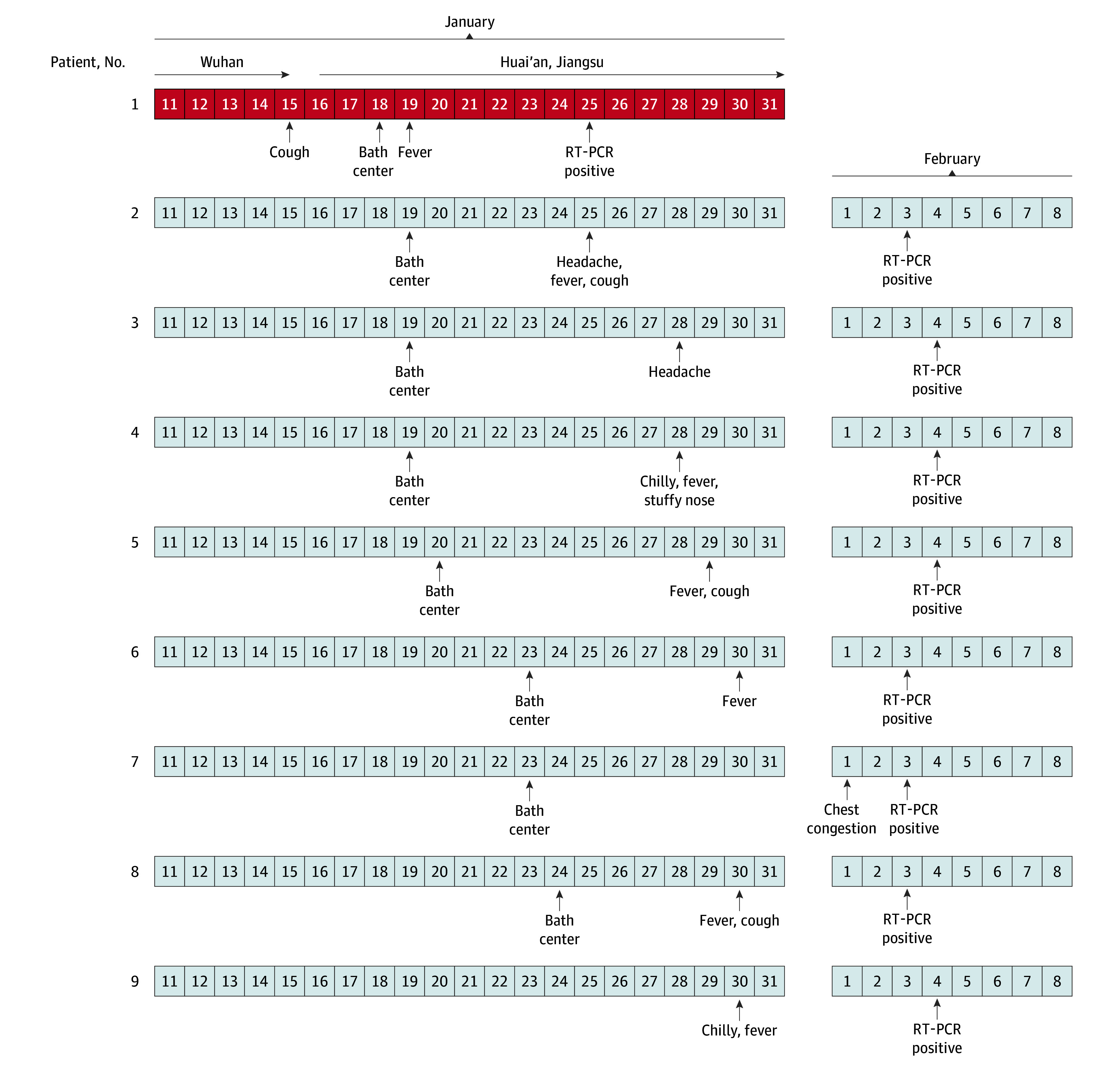
Chronology of Onset Date and Diagnosis Information of the Cluster Patient 1 traveled from Wuhan, China, to Huai’an, Jiagnsu Province, China, and the dates of onset and diagnosis for this patient appear in red. Patient 9 was an indirect contact worker in the bath center. RT-PCR indicates reverse transcription–polymerase chain reaction assay.

The median (interquartile range) age of the patients was 35 (24-50) years. A total of 8 patients (89%) reported fever (mean [SD] duration, 5.78 [2.99] days), and 7 patients (78%) reported a cough. Few patients (3 [33%]) showed debilitation, chest distress (2 [22%]), or anorexia (1 [11%]). Diarrhea, myalgia, rhinorrhea, and headache were not reported. C-reactive protein levels were elevated in 9 patients (100%; mean [SD], 3.34 [3.18] mg/dL; to convert to milligrams per liter, multiply by 10). Lymphopenia occurred in 3 patients (33%), lactate dehydrogenase was increased in 3 patients (33%; mean [SD], 225.56 [85.33] U/L; to convert to microkatals per liter, multiply by 0.0167), and glutamic oxaloacetic transaminase was increased in 2 patients (22%; mean [SD], 30.22 [13.94] U/L) (Table).

**Table.** Clinical Presentation and Pertinent Laboratory Findings of 9 Patients Who Visited the Same Bath Center and Were Diagnosed With Coronavirus Disease 2019

Characteristic	Data
Age, median (IQR), y	35 (31-43)
Sex, No. (%)	
Men	9 (100)
Women	0
Febrile days, mean (SD)	5.78 (2.99)
Maximum temperature, mean (SD), °C	38.11 (0.53)
Cough, No. (%)	7 (78)
Days of cough, mean (SD)	5.33 (4.15)
Debilitation, No. (%)	3 (33)
Chest distress, No. (%)	2 (22)
Anorexia, No. (%)	1 (11)
Diarrhea, No. (%)	0
Myalgia, No. (%)	0
Rhinorrhea, No. (%)	0
Headache, No. (%)	0
Laboratory results, mean (SD)	
CRP, mg/dL	3.34 (3.18)
White blood cells, /μL	5470 (1970)
Red blood cells, ×10^6^/μL	5.20 (0.41)
Hemoglobin, g/dL	15.92 (1.15)
Hematocrit, %	45.38 (3.32)
Platelets, ×10^3^/μL	155.67 (67.80)
Neutrophils, %	66.01 (9.86)
Absolute neutrophils, /μL	3680 (1490)
Lymphocytes, %	23.66 (8.90)
Absolute lymphocytes, /μL	1230 (460)
Monocytes, %	8.93 (1.83)
Absolute monocytes, /μL	460 (110)
Eosinophils, %	1.3 (2.52)
Absolute eosinophils, /μL	150 (250)
Basophils, %	0.11 (0.08)
Absolute basophils, /μL	5 (5)
Procalcitonin, ng/mL	0.16 (0.07)
Creatine kinase, U/L	93.56 (36.22)
Glutamic oxaloacetic transaminase, U/L	30.22 (13.94)
Lactate dehydrogenase, U/L	225.56 (85.33)

Abbreviations: CRP, C-reactive protein; IQR, interquartile range.

SI conversion factors: To convert absolute basophils, absolute eosinophils, absolute lymphocytes, absolute monocytes, and absolute neutrophils to ×10^9^ per liter, multiply by 0.001; basophils, eosinophils, lymphocytes, monocytes, and neutrophils to proportion of 1, multiply by 0.01; CRP to milligrams per liter, multiply by 10; creatine kinase to microkatals per liter, multiply by 0.0167; hematocrit to proportion of 1.0, multiply by 0.01; hemoglobin to grams per liter, multiply by 10; lactate dehydrogenase to microkatals per liter, multiply by 0.0167; platelets to ×10^9^ per liter, multiply by 1.0; red blood cells to ×10^9^ per liter, multiply by 1.0; and white blood cells to ×10^9^ per liter, multiply by 0.001.

Computed tomography examinations were performed, and the ground glass opacities were observed in all 9 patients. As of February 10, 2020, no patients required respiratory support.

## Discussion

Previous studies have demonstrated that the transmission rate of a virus is significantly weakened in an environment with high temperature and humidity.^4^ However, judging from the results of this study, the transmissibility of SARS-CoV-2 showed no signs of weakening in warm and humid conditions. We noticed a clustered case occurring in a public bath center with high temperature and humidity. A total of 8 individuals who used or worked in the bath center experienced symptoms within 6 to 9 days of their visit to the center, suggesting that SARS-CoV-2 could spread and cause infection in such an environment. The transmission routes may also be respiratory droplets or contact, but our results suggest that the cluster transmission of SARS-CoV-2 can still arise in an environment with high temperature and humidity. These results provide a potential epidemiological clue for this novel coronavirus. This study was limited by a lack of detail regarding the transmission routes of the patients in the bath center.
