# Error in Funding/Support

**DOI:** 10.1001/jamanetworkopen.2020.17413

**Published:** 2020-08-05

In the Original Investigation titled “Possible Transmission of Severe Acute Respiratory Syndrome Coronavirus 2 (SARS-CoV-2) in a Public Bath Center in Huai’an, Jiangsu Province, China,”^1^ published March 30, 2020, there was an error in the Funding/Support section in the Article Information. Dr Zhang received award number 81800149. This article has been corrected.^1^
